# Clinical characteristics of post-traumatic epilepsy and the factors affecting the latency of PTE

**DOI:** 10.1186/s12883-021-02273-x

**Published:** 2021-08-05

**Authors:** Tingting Yu, Xiao Liu, Lei Sun, Jianping Wu, Qun Wang

**Affiliations:** 1 Department of Neurology, Beijing Tiantan Hospital, Capital Medical University, Beijing, 100070 P. R. China; 2 China National Clinical Research Center for Neurological Diseases, Beijing, 100070 P. R. China; 3 Advanced Innovation Center for Human Brain Protection, Capital Medical University, Beijing, 100070 P. R. China; 4 Beijing Institute for Brain Disorders, Beijing, 100069 P. R. China

**Keywords:** Post-traumatic epilepsy, Traumatic brain injury, Clinical characteristics, Latency, Risk factors

## Abstract

**Objectives:**

To summarize the clinical characteristics of post-traumatic epilepsy (PTE), and to identify the factors affecting the latency of PTE after traumatic brain injury (TBI).

**Methods:**

We conducted a retrospective clinical analysis in patients with PTE who visited the outpatient Department of Epilepsy, Beijing Tiantan Hospital from January 2013 to December 2018. The clinical characteristics, including gender, age distribution, seizure type, and latency were summarized. Factors affecting the latency of PTE were evaluated using Kaplan-Meier curves and Cox proportional hazard regression analysis.

**Results:**

Complete clinical information was available for 2862 subjects, of which 78.48% were males. The mean age at TBI was 21.4 ± 15.1 years and peaked in the 0 to 12-year-old and 15 to 27-year-old groups. Generalized onset seizure was the most frequent seizure type (72.82% of patients). Approximately 19.95% PTE patients developed drug-resistant epilepsy. The latency of PTE ranged from 8 days to 20 years, with a median of 24.0 (IQR, 5.0–84.0) months. The Kaplan-Meier curves demonstrated that gender, age at TBI, severity of TBI, multiple craniocerebral injuries, post-TBI treatments, acute seizures, and residual disability were associated with PTE latency. The Cox regression model indicated that age ≥ 18 years old, severe TBI with multiple surgical operations, acute seizures, and residual disability were risk factors for shorter PTE latency.

**Conclusions:**

PTE is more common in males than females, and peaked in the 0 to 12-year-old and 15 to 27-year-old groups. Generalized onset seizure was the most common seizure type and 19.95% of participants developed drug-resistant epilepsy. Patients aged ≥18 years old, who suffered severe TBI followed by multiple surgical operations, experienced acute seizures, or with residual disabilities had shorter PTE latency.

## Background

Traumatic brain injury (TBI) is a common public health concern. More than 50 million new cases of TBI are reported each year worldwide [1], with nearly a million cases occurring in China [2]. Post-traumatic epilepsy (PTE) is one of the most common and disabling sequela of TBI, defined as repeated unprovoked seizures seven days after TBI. The incidence of PTE in the civilian population following TBI is 2 to 17%, and is correlated with the severity of TBI (mild TBI: 2.1%; moderate TBI: 4.2%; severe TBI: 16.7%) [3–8]. Among military patients with penetrating TBI, the incidence of PTE is significantly higher, at 22 to 53% [6]. In terms of composition ratio, PTE accounts for 5% of epilepsy cases and 20% of symptomatic epilepsy cases [6, 9].

For a long time, many published studies have focused on risk factors for PTE. Moderate-to-severe TBI, males, post-traumatic amnesia, focal neurologic signs, loss of consciousness at initial TBI, abnormal neuroimaging findings, acute seizures (defined as seizures occurring within seven days after TBI), and age ≥ 65 years have been reported as risk factors for PTE. Meanwhile, the more serious the TBI is, the longer the risk increases [3, 5, 10, 11]. However, those studies mainly followed up with select TBI patients, in which the number of cases of PTE was small, and there was a lack of systematic description of PTE clinical characteristics.

Early prophylactic antiepileptic drug (AED) therapy after TBI, which can reduce the onset of acute seizures, has not been shown to provide protective effects against the onset of later PTE [12–14]. There is a period of latency between the initial TBI and the onset of seizures, and if the latency can be prolonged, the PTE process can be delayed thus reducing the patient’s disease and the resulting financial burden [11]. Unfortunately, few studies have focused on the latency of PTE and, as such, our understanding of latency remains insufficient [5, 15, 16]. To address this gap in our understanding of PTE latency and the limitations of previous studies, the current study sought to summarize the characteristics of PTE and to identify factors affecting PTE latency.

## Methods

### Study participants

This study was designed as a retrospective study. We collected data from the outpatient electronic medical record system of patients diagnosed with PTE at the Epilepsy Center of Beijing Tiantan Hospital from January 2013 to December 2018. Inclusion criteria included: (1) Diagnosed with “PTE” in the outpatient department; (2) Willing to be followed up, and; (3) Possessing complete medical records. Exclusion criteria included: (1) Does not meet the diagnostic criteria of PTE after evaluation (e.g., only one seizure occurred after TBI); (2) Presence of perinatal injury, febrile convulsion, or previous seizure; (3) Pre-existing neurological disease; (4) TBI ≥ twice before first-time seizure, or; (5) First-time seizure onset > 20 years after TBI.

The study was approved by the Ethics committee of the Beijing Tiantan Hospital affiliated with the Capital Medical University of the People’s Republic of China. The study was conducted in accordance with the Declaration of Helsinki, and all participants provided informed consent for the use of their medical records.

### Data collection

“The PTE patient information registration form” was designed to collect data, including demographic information, family history, personal medical records, details of the TBI, clinical condition of PTE, and auxiliary examinations. It was filled out through case review, face-to-face interviews, or telephone follow-up by formally trained neurologists. To ensure the clinical observation of PTE was greater than 1 year, all forms were filled out at least 12 months after first-time late post-traumatic seizure (LPTS) onset.

The auxiliary examinations included mainly electroencephalogram (EEG), cranial computed tomography (CT) and/or cranial magnetic resonance imaging (MRI). The first-time EEG examination, CT and/or MRI after TBI was preferentially recorded. If missing, the last auxiliary examination result was recorded. For the majority of cases, original EEG and/or imaging data were reviewed. For a minority of cases, the EEG and/or imaging data were recorded according to the findings documented in the medical records as we failed to find the original data.

### Concept definitions

Previous studies have suggested that the effects of TBI on PTE may persist for up to 15–20 years, while the risk of PTE in patients with TBI compared to the general population showed no significant difference 15–20 years later after TBI [3, 5]. Therefore, we believe that the effect of TBI on seizures was inconclusive for those who had first-time seizure onset more than 20 years after TBI, so they were excluded.

TBIs were divided into three categories of severity based on neurological and imaging evaluation as follows: TBI without skull fracture and with loss of consciousness or post-trauma amnesia lasting < 30 min was considered “mild”; TBI with loss of consciousness or post-trauma amnesia lasting 30 min to < 24 h, or with skull fracture was considered “moderate”; TBI with brain contusion, intracranial hematoma, loss of consciousness, or post-trauma amnesia lasting ≥24 h was considered “severe” [3] .

Craniocerebral injury was assessed according to the site of lesion caused by the TBI. Single injury referred to a single or continuous lesion (e.g., unilateral frontotemporal, temporal parietal), while multiple injuries referred to lesions of bilateral involvement or topographically separate locations (e.g., left frontal and right occipital, bilateral frontal).

Seizure type was divided into generalized or focal onset seizure according to the 2017 classification of the International League Against Epilepsy (ILAE) [17]. We defined the most frequent seizures within the last calendar year for each subject as his/her seizure type.

Drug-resistant epilepsy was defined as “failure of achieving a seizure-free duration of 3 times the interseizure interval or 1 year (depending on which is longer) of two tolerated, appropriately chosen and used AEDs (whether as monotherapies or in combination)” [18].

The latency of PTE was defined as the time interval between the onset of first-time LPTS and TBI.

### Statistical analysis

We used Microsoft Excel to build a database and SPSS 23.0 software (IBM Crop., Armonk, NY) for data analysis. Measurement data were represented by mean ± standard deviation (SD) or median, and enumeration data were expressed as a percentage. A chi-square test was used to analyze the clinical characteristic of patients that had different types of seizure. Kaplan-Meier curves and a Log-rank test were used to analyze the latency distribution in variables. A Cox proportional hazard regression model was used to examine relationships between variables and survival endpoint. The endpoint was defined as the onset of first-time LPTS. Latency (in months) was used to compute the time variable. Predictive variables included gender, age at TBI, family history of epilepsy, severity of TBI, single or multiple injuries, post-TBI treatments, acute seizures, residual disability, EEG results, and neuroimaging results.

## Results

### PTE characteristics

#### Demographic information

**Fig. 1:**
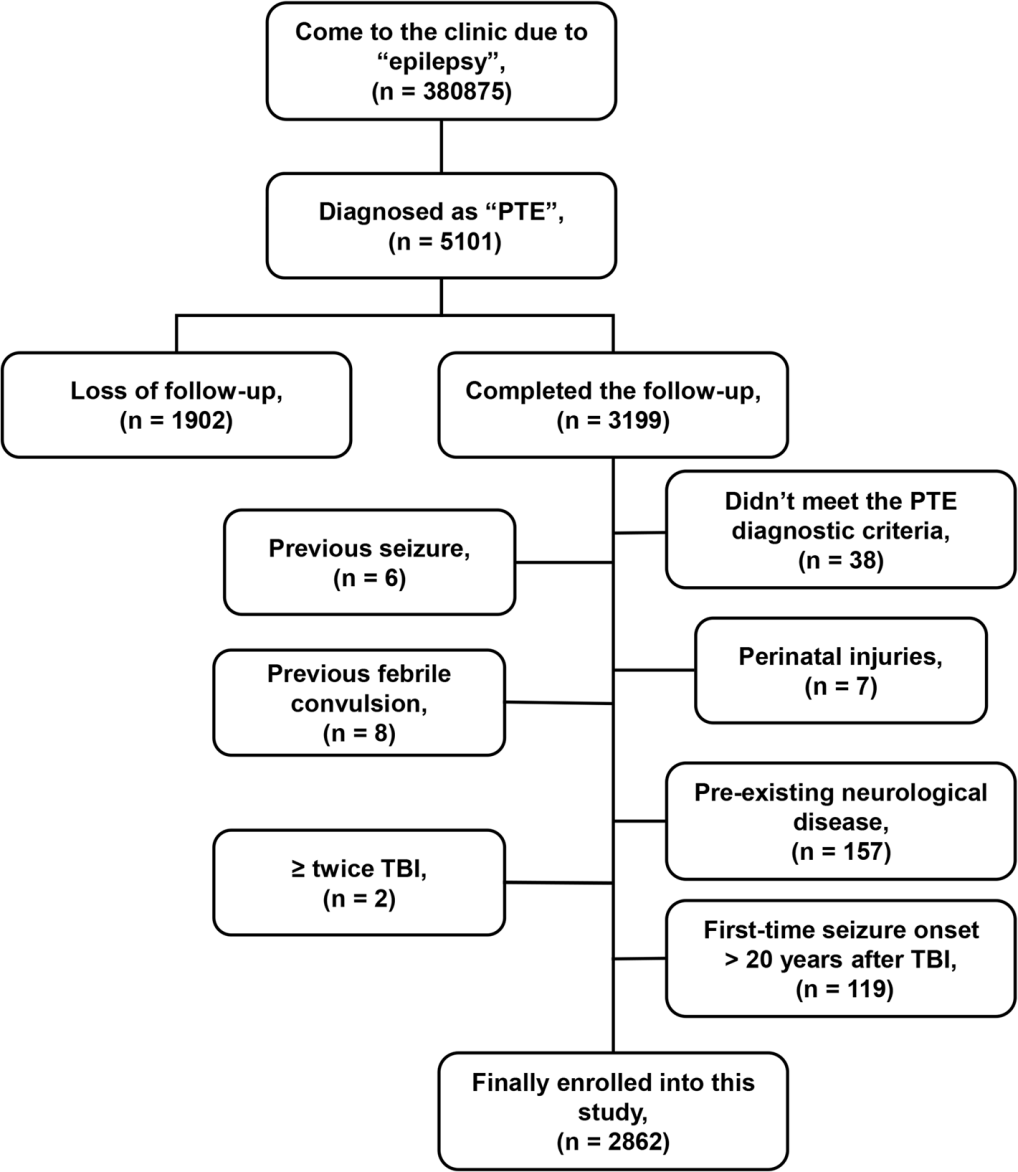
Case screening flow diagram. A total of 380,875 patients came to the outpatient Department of Epilepsy, Beijing Tiantan Hospital from January 2013 to December 2018, of which 5101 patients were diagnosed with PTE. Among them, 1902 patients were lost to follow-up, and 3199 patients completed the follow-up and the information registration form. After screening, 337 of the 3199 patients were excluded, and 2862 patients were finally enrolled in this study

A total of 380,875 patients visited the outpatient Department of Epilepsy at the Beijing Tiantan Hospital from January 2013 to December 2018. Among them, 5101 patients were diagnosed with “PTE”, and 3199 of them completed the follow-up and questionnaire. Finally, we excluded 337 patients and 2862 were enrolled in this study. Our cohort design is depicted in the flow diagram in Fig. 1.

**Fig. 2:**
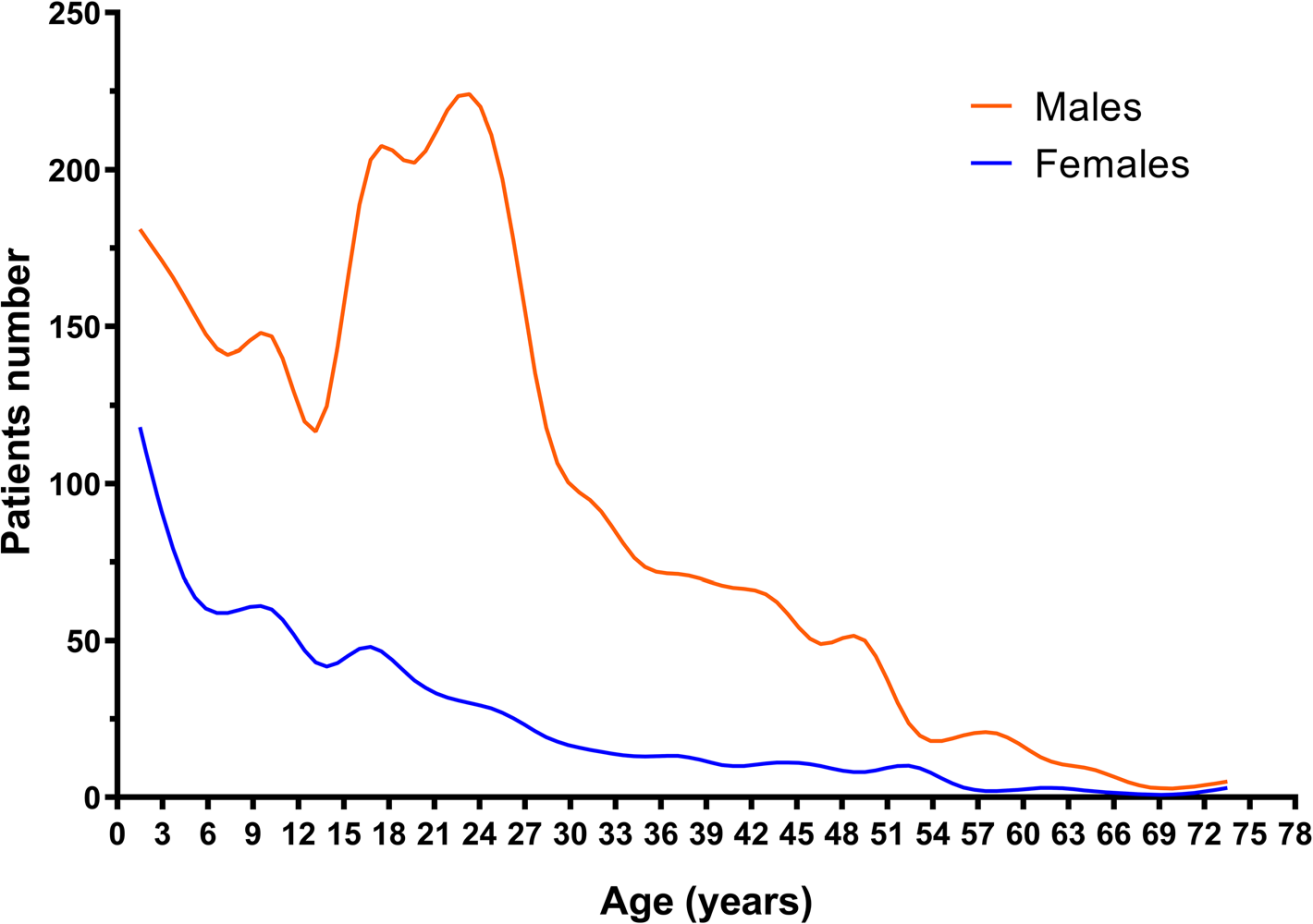
Gender and age distribution of study participants. The age of TBI occurrence ranged from birth to 86.0 years, peaked in 0–12 (931/2862) and 15–27 years (1023/2862): Males peaked in 0–12 (626/2246) and 15–27 years (867/2246), females peaked in 0–12 years (305/616)

Among all study participants, 2246 were males, and the male to female ratio was 3.6: 1. The age at TBI ranged from birth to 86.0 years, with a mean age of 21.4 ± 15.1 years. A total of 2192 participants (76.6%) suffered TBI at the age of 0–30 years, with a peak between 0 to 12 years (931/2862) and 15 to 27 years (1023/2862). The age of males peaked in 0–12 (626/2246) and 15–27 years (867/2246), while females only peaked in 0–12 years (305/616) (Fig. 2). A clear family history of epilepsy was observed in 10 participants (0.35%).

#### Details of TBI and post-TBI treatment

Following TBI evaluation, 203 (7.09%) participants had mild TBI, 1180 (41.23%) participants had moderate TBI, and 1479 (51.68%) patients had severe TBI. Additionally, 1427 (49.86%) participants had single craniocerebral injury, and 1435 (50.14%) participants had multiple craniocerebral injuries.

Participants received different treatments after TBI depending on the severity of their injury (Table 1). All the participants with mild TBI, 1176 (99.7%) participants with moderate TBI, and 483 (32.7%) participants with severe TBI received conservative treatments, while 3 (0.3%) participants with moderate TBI and 604 (40.8%) participants with severe TBI underwent one surgical operation of puncture drainage or decompressive craniectomy (PD/DC) during the acute phase of TBI, and 1 (0.1%) participant with moderate TBI and 392 (26.5%) participants with severe TBI underwent multiple surgical operations, including one PD/DC operation during the acute phase and another cranioplasty (CP) operation later.

**Table 1:** Post-TBI treatments of patients of mild, moderate, and severe TBI

	Conservative treatment (%)	PD/DC (%)	PD/DC + CP (%)
Mild TBI	203 (100)	0	0
Moderate TBI	1176 (99.7)	3 (0.3)	1 (0.1)
Severe TBI	483 (32.7)	604 (40.8)	392 (26.5)
*P =* 0.000^**^			

*PD* puncture drainage, *DC* decompressive craniectomy, *CP* cranioplasty

^**^P < 0.01

After treatment and rehabilitation, 2547 (88.99%) participants had a Modified Rankin Scale score (mRS) of 0 to 2, assessed as without residual disability 6 months after TBI, and 315 (11.01%) participants had an mRS score of 3 to 5, assessed as with residual disability 6 months after TBI.

#### Post-traumatic seizure

Following TBI, 171 (5.97%) patients experienced acute seizures. As for LPTS, 2084 (72.82%) participants experienced generalized onset seizures as the most frequent type of seizure, whereas 495 (17.30%) participants experienced focal onset seizures as the most frequent type of seizure. We could not identify the most frequent type of seizure in 283 (9.89%) participants because they had similar frequency of both seizure types. Participants who were males, ≥ 18 years old, had moderate to severe TBI, had single craniocerebral injuries, with residual disability, with normal neuroimaging were more likely to develop generalized onset seizure (Table 2). Additionally, 571 (19.95%) participants developed drug-resistant epilepsy.

**Table 2:** Clinical characteristics of patients had different types of seizure

Factors	Generalized onset	Focal onset or both onset	***P***-Value
Gender			0.009*
Males	1661 (74.0)	585 (26.0)	
Females	423 (68.7)	193 (31.3)	
Age at TBI (years)			0.000**
< 18	863 (69.5)	379 (30.5)	
≥ 18	1221 (75.4)	399 (24.6)	
Family history of epilepsy			0.148
No	2079 (72.9)	773 (27.1)	
Yes	5 (50.0)	5 (50.0)	
The severity of TBI			0.000**
Mild	123 (60.6)	80 (39.4)	
Moderate	882 (74.7)	298 (25.3)	
Severe	1079 (73.0)	400 (27.0)	
Craniocerebral injury			0.000**
Single	1122 (78.6)	305 (21.4)	
Multiple	962 (67.0)	473 (33.0)	
Post-TBI treatment			0.233
Conservative treatment	1343 (72.1)	519 (27.9)	
PD/DC	441 (72.7)	116 (27.3)	
PD/DC + CP	300 (76.3)	93 (23.7)	
Acute seizure			0.331
Without	1954 (72.6)	737 (27.4)	
With	130 (76.0)	41 (24.0)	
Residual disability			0.020*
Without	1872 (73.5)	675 (26.5)	
With	212 (67.3)	103 (32.7)	
EEG findings ^a^			0.085
Normal	100 (72.5)	38 (27.5)	
Unnormal background	56 (71.8)	22 (28.2)	
Epileptiform discharges	262 (63.4)	151 (36.6)	
Cranial CT/MRI findings ^b^			0.005**
Normal	325 (84.4)	60 (15.6)	
Abnormal	509 (77.1)	151 (71.6)	

*PD* puncture drainage, *DC* decompressive craniectomy, *CP* cranioplasty

* *P* < 0.05; ** *P* < 0.01

^a^ Data from only 629 patients

^b^ Data from only 1045 patients

#### Auxiliary examinations

As shown in Table 3, EEG data were successfully collected from 629 patients. The abnormal rate of EEG was 78.06%. Normal findings were observed in 138 participants, abnormal background waves without epileptiform discharges was observed in 78 participants, and epileptiform discharges were observed in 413 participants. Of the 413 participants with epileptiform discharges, 156 (37.8%) had diffuse epileptiform discharges and 257 (62.2%) had regional epileptiform discharges. Regional epileptiform discharges were most common in the temporal lobe (68.09%), including in 54 participants without temporal lobe lesions.

**Table 3:** EEG results of 629 patients

Results	No.	Proportion (%)
Normal	138	21.94
Abnormal background waves	78	12.40
Epileptiform discharges	413	65.66
Left temporal lobe	96	15.26
Right temporal lobe	79	12.56
Other localisation	82	13.04
Diffuse epileptiform discharges	156	24.80

Neuroimaging results were successfully collected from 1045 participants, with 385 participants showing normal findings, and 660 participants showing abnormal findings (Table 4).

**Table 4:** Cranial CT/MRI results of 1045 patients

Results	No.	Proportion (%)
Normal	385	36.84
Encephalomalacia foci	234	22.39
Medial temporal lobe atrophy	61	5.84
Fractures	53	5.07
Enlarged lateral ventricular	30	2.87
Intracranial hemorrhage	11	1.05
Brain contusion	7	0.67
Multiple abnormalities	264	25.26

### The latency of PTE and the factors affecting it

#### The latency of PTE

**Fig. 3:**
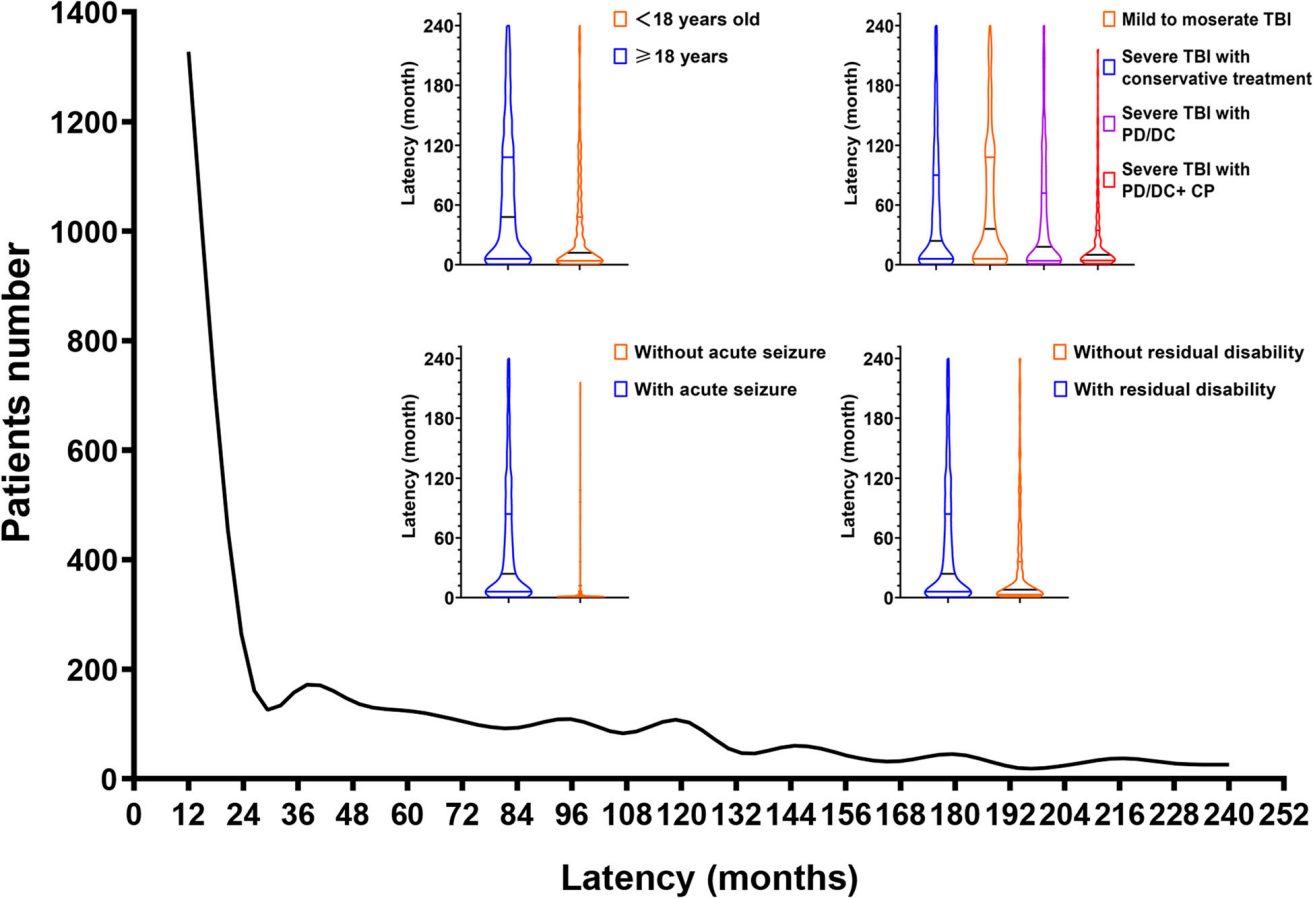
The distribution of PTE latency. The latency of PTE ranged from 8 days to 20 years, with a median latency of 24.0 (IQR, 5.0–84.0) months. The latency of PTE lasted 8 days to 6 months in 32.70% (936/2862) patients, 7 to 12 months in 13.66% (391/2862) patients, 13 to 24 months in 9.36% (243/2862) patients, 24 to 60 months in 14.99% (429/2862) patients, 61 to 120 months in 17.37% (497/2862) patients, and longer than 120 months in 12.79% (366/2862). The violin plot shows that patients who had TBI at the age ≥ 18 years old, suffered severe TBI, underwent multiple surgical operations CP operation after severe TBI, experienced acute seizures, and had residual disability tend to have a shorter latency

The latency of PTE ranged from 8 days to 20 years, with a median of 24.0 (IQR, 5.0–84.0) months. The latency of PTE lasted from 8 days to 6 months in 936 participants (32.7%), 7 to 12 months in 391 participants (13.7%), 13 to 24 months in 243 participants (8.5%), 24 to 60 months in 429 participants (15.0%), 61 to 120 months in 497 participants (17.4%), and longer than 120 months in 366 participants (12.8%) (Fig. 3).

#### Factors affecting the latency of PTE

The results of the univariate analyses are shown in Table 5. There were statistically significant differences in the distribution of latency between the following variables: gender (*P* = 0.080), age at TBI (*P* < 0.001), the severity of TBI (*P* = 0.003), single or multiple craniocerebral injuries (*P* = 0.050), post-TBI treatment (*P* < 0.001), acute seizure (*P* < 0.001), and residual disability (*P* < 0.001). None of the following variables resulted in a significant difference in the distribution of PTE latency: family history of epilepsy, abnormal EEG results, or abnormal neuroimaging results.

**Table 5:** Results of Log-rank test of latency distribution in variables

Variables	No. (%)	Latency (IQR) (mo)	χ2	***P***-Value
Gender			6.998	0.080*
Males	2246 (78.48)	23.0 (5.0–72.0)		
Females	616 (21.52)	36.0 (6.0–96.0)		
Age at TBI (years)			140.533	0.000**
< 18	1242 (43.40)	48.0 (6.0–108.0)		
≥ 18	1620 (56.60)	12.0 (4.0–48.0)		
Family history of epilepsy			0.898	0.343
No	2852 (99.65)	24.0 (5.0–84.0)		
Yes	10 (0.35)	24.0 (22.0–108.0)		
The severity of TBI			11.743	0.003**
Mild	203 (7.09)	48.0 (6.0–84.0)		
Moderate	1180 (41.23)	24.0 (5.0–90.0)		
Severe	1479 (51.68)	17.0 (5.0–72.0)		
Craniocerebral injury			7.955	0.005**
Single	1427 (49.86)	24.0 (6.0–84.0)		
Multiple	1435 (50.14)	19.0 (4.0–72.0)		
Post-TBI treatment			75.998	0.000**
Conservative treatment	1862 (65.06)	24.0 (6.0–96.0)		
PD/DC	607 (21.21)	18.0 (4.0–72.0)		
PD/DC + CP	393 (13.73)	10.0 (4.0–33.0)		
Acute seizure			315.064	0.000**
Without	2691 (94.03)	24.0 (6.0–84.0)		
With	171 (5.97)	1.0 (1.0–3.0)		
Residual disability			36.876	0.000**
Without	2547 (88.99)	24.0 (6.0–84.0)		
With	315 (11.01)	8.0 (3.0–36.0)		
EEG findings ^a^			0.935	0.627
Normal	138 (21.94)	36.0 (6.0–96.0)		
Abnormal background	78 (12.40)	12.0 (4.0–72.0)		
Epileptiform discharges	413 (65.66)	34.0 (6.0–84.0)		
Cranial CT/MRI findings ^b^			0.649	0.421
Normal	385 (36.84)	24.0 (5.0–84.0)		
Abnormal	660 (63.16)	18.0 (3.0–84.0)		

*IQR* interquartile range, *PD* puncture drainage, *DC* decompressive craniectomy, *CP* cranioplasty

* *P* < 0.10; ** *P* < 0.01

^a^The data from only 629 patients

^b^The data from only 1045 patients

To identify factors that independently affect PTE latency, variables that resulted in a significant difference using the Log-rank test were entered into a Cox regression analysis. The results of the univariate analysis showed that there was no significant difference in the distribution of latency between participants with mild TBI and participants with moderate TBI (*P* = 0.740). When constructing the proportional hazard model in the multivariate analysis, we combined mild and moderate TBIs together for analysis. Additionally, participants with severe TBI were stratified into three subgroups (severe TBI with conservative treatment, severe TBI with PD/DC, severe TBI with PD/DC + CP) based on their post-TBI treatments. The results showed that age ≥ 18 years, severe TBI followed by multiple surgical operations, presence of acute seizures, and residual disability after TBI were risk factors for shorter latency, while severe TBI followed by conservative treatments was a protective factor. Gender and single or multiple craniocerebral injuries were not independent factors affecting PTE latency (Table 6).

**Table 6:** Results of Cox proportional hazard model for PTE latency in 2862 PTE patients

	HR	HR95%***CI***	***P***-Value
Females	0.969	0.883 - 1.062	0.498
≥ 18 years old	1.527	1.412 - 1.650	0.000^**^
The severity of TBI			
Mild & moderate TBI	1		
Severe TBI with conservative treatment	0.879	0.792 - 0.976	0.016^*^
Severe TBI with PD/DC	0.997	0.904 - 1.099	0.945
Severe TBI with PD/DC + CP	1.293	1.149 - 1.455	0.000^**^
Multiple craniocerebral injuries	1.045	0.969 - 1.127	0.254
With acute seizures	3.635	3.100 - 4.262	0.000^**^
With residual disability	1.381	1.225 - 1.557	0.000^**^

*PD* puncture drainage, *DC* decompressive craniectomy, *CP* cranioplasty

^*^P < 0.05; ^**^P < 0.01

## Discussion

PTE is one of the most common and serious complications of TBI, leading to poor functional outcomes and a medical burden for survivors of TBI [19, 20]. There is a lack of investigations on the clinical characteristics and latency of PTE, especially with a large sample size of patients with PTE. The current study enrolled 2862 participants diagnosed with PTE, summarized the clinical characteristics of PTE, and found that age at TBI, severity of TBI, post-TBI treatments, acute seizures, and residual disability were independent factors affecting the latency of PTE, thereby providing a reference point for survivors of TBI when making therapeutic decisions.

### Clinical characteristics of PTE

Comparing the gender composition of the study participants, we found that males are significantly more likely than females to be diagnosed with TBI, as has been reported [4, 21]. Males and females have different personality traits and gender roles: males are more aggressive, are involved in a wider range of social activities, and are more susceptible to TBI, especially young adult males [2]. Several studies have also indicated that being male was an independent risk factor for PTE after TBI [8, 10]. This might be related to the hormonal differences between the sexes [22]. Higher rates of alcohol abuse among males might also play a role [10]. However, we lacked the data on alcohol use for further analysis. The age range with the most cases of TBI peaked in the 0 to 12 and 15 to 27 year-old groups, consistent with previous studies on PTE in children [5] and adults [8]. The aggregation of patients in the 0 to 12 year-old group might be related to the strong sense of curiosity and lack of discernment amongst children.

Generalized onset seizures were the most common seizure type, accounting for 72.8% of seizures, which is consistent with the previously reported rate of 79.0% [4]. This might be attributed to the altered brain microenvironment after TBI because of decreased cerebral blood flow, altered metabolism, increased neuronal excitability, and large amounts of hemosiderin deposited in the neural fiber network. One the contrary, Tubi et al. reported that continuous EEG monitoring indicated more than half of seizures after TBI were focal onset, and 20 to 30% of what we think of as generalized tonic-clonic seizures were actually focal to bilateral tonic-clonic seizures [20, 23]. Unfortunately, EEG during epileptic seizures was not collected in all participants, thus we were not able to verify the seizure type based on EEG. We also observed that demographic characteristics and TBI details might be factors that impact the development of generalized onset or focal onset seizures.

### PTE latency and factors affecting the latency

#### The latency of PTE

We found that the proportion of patients who had a PTE latency period shorter than 1 year was lower than what Zhao and Englander reported [4, 7], but was consistent with other studies which included a larger number of participants and had longer follow-up periods [3, 5, 15]. In addition, a study of PTE in adults indicated that the median PTE latency period was 1 year after 10 years of follow-up [8], and in this study we found the median PTE latency period was 2 years. Considering that previous studies might miss patients who developed first-time seizure after the follow-up period due to limited follow-up time, we believe that our results on the latency are relatively reliable, as this study was not limited by the follow-up time and no PTE patients would be missing.

#### Factors affecting the latency of PTE

In this study, participants who suffered TBI at age ≥ 18 years old had shorter latency than those who suffered TBI at age < 18 years old. Christensen et al. [5] also reported that TBI at age ≥ 15 years old was an independent risk factor for PTE. Similar observations of older age on PTE has been reported by Annegers [3] and Zhao [7]. Those studies suggested that epilepsy susceptibility after TBI increases with age, and might be associated with neuroinflammation, decreased neuronal metabolism, neuronal degeneration, and abnormal cerebral hemodynamics [24]. Interestingly, epileptic discharges in juveniles were more frequent than in adults during the acute phase of TBI. A multicenter study reported that epileptic discharges were observed in 42.5% of TBI cases in children, and younger age was a significant risk factor for post-traumatic seizure (PTE) and status epilepticus during the acute phase [25]. The differences in the risk of epileptic seizures between adult and juvenile patients at different periods after TBI are related to the characteristics of brain development of patients of different ages: the cerebral cortex of juvenile patients is immature, the function of inhibiting nerve reflex is not established yet, and they are more sensitive to external injury stimuli. Therefore, abnormal discharges of cerebral neurons are more likely to occur in the acute phase of TBI for juvenile patients, which presents as sub-clinical epileptic discharge or acute symptomatic seizures. At the same time, the young brain of juvenile patients is more malleable and adaptable than the aging brain, so it is less likely to form a chronic epileptic brain network after the acute phase of TBI.

The severity of TBI is an established etiological risk factor for PTE [3, 5, 6, 10]. It was reported that patients who had more severe TBI may develop recurrent seizures within a shorter time interval and may have more frequent seizures [23]. In terms of the distribution of PTE latency, we found that there was no difference between participants who had mild TBI and moderate TBI. PTE latency in participants that suffered severe TBI was related to their post-TBI treatments: latency in participants who received conservative treatments was longer than that of participants who had mild to moderate TBI; the latency of participants that underwent a single surgical operation (PD/DC) showed no difference with that of participants that had mild to moderate TBI; and the latency of patients undergoing multiple surgical operation (PD/DC + CP) was shorter than that of participants that had mild to moderate TBI. This observation is not only related to the brain damage caused by TBI, but also to the secondary brain damage caused by the operation itself. As seizures are mainly related to abnormal discharges of the cerebral cortical network, patients with severe TBI are more likely to have damage in the deep brain and even the brain stem rather than the cerebral cortex or subcortical, which might explain why they have a longer latency. However, surgical procedures after TBI can cause significant damage to the cerebral cortex, especially the CP procedure. For patients with TBI complicated with intracranial edema, cerebral hernia, or high cranial pressure, PD/DC surgery helps to expand brain volume, reduce intracranial pressure, and reduce mortality after TBI. Thus, it is recommended that the PD/DC procedure be performed as soon as possible after TBI for those who meet the surgical indication. Although CP might shorten the latency, some reports have shown that it plays an important role in regulating brain blood flow, improving brain metabolism, and reducing the complications caused by PD/DC surgery. Additionally, CP could effectively eliminate the abnormal appearance of bone flap defects, and reduce the psychological burden of patients with TBI [26]. Therefore, we suggest that a multidisciplinary assessment be made to make recommendations regarding the decision and timing of CP operation. While it has been reported that the high incidence of PTE may be related to multiple craniocerebral injuries and lesion location (the temporal lobe) [4, 20], we found that latency was not affected by single or multiple craniocerebral injuries.

Annegers et al. [3] reported that acute seizures were not a risk factor for PTE. Alan et al. [23] also reported that the seizure recurrence rate did not increase in patients with acute seizures. On the contrary, other studies [4, 7] found that acute seizures were a predictor of PTE, and the secondary brain injury caused by acute seizures plays an important role in PTE progression [27, 28]. The results of our study support the latter view as we found that patients with acute seizures had shorter latency. Thus, we recommend that prophylactic AED treatments be administered in the acute phase of TBI. Although the incidence of PTE is not reduced, it might reduce acute seizures, which is expected to prolong PTE latency by reducing secondary brain injury, thus improving the prognosis of PTE [13].

We found that patients who had residual disability after TBI had a significantly shorter PTE latency than those who did not have residual disability, which was not described in previous studies. We believe that short PTE latency and residual disability might be mutually reinforcing, but further investigation is required to confirm this hypothesis.

Continuous EEG monitoring during the acute phase helps to identify subclinical epileptic discharges and non-convulsive epileptic state [25]. While epileptiform abnormalities have been reported to increase the risk of PTE, especially sporadic epileptiform [29], it showed no effect on PTE latency in this study. We observed an abnormal EEG rate of 78.0%, lower than the previous reported rate of 90.0% [7]. The discrepancy in abnormal EEG rate may due to the fact that EEG examination was performed during the interictal stage in most patients in this study, and the EEG monitoring time might have been too short to detect abnormalities.

We found that the neuroimaging abnormalities also did not affect PTE latency, in contrast with its effect on the incidence of PTE [3, 5, 7, 8]. This may be related to the fact that not all the neuroimaging was performed within 24 h after TBI. That is to say, the neuroimaging results might not truly reflect the changes in craniocerebral structure in the acute phase of TBI. Further studies are needed to determine the exact effects of craniocerebral structural damage on PTE latency.

### Limitation

As a retrospective study, this study is limited by the inherent to the retrospective nature. Most of the participants in the study were from Beijing and surrounding areas, the results might not be representative of the general situation around China. The results of long-tern EEG monitoring and neuroimaging during the acute phase of TBI were lacking in a part of subjects. Therefore, partial description of the clinical characteristics of PTE lacks of sufficient data support. In addition, this study didn’t analyze all reported risk factors for PTE (e.g., alcoholism, post-traumatic amnesia, focal neurologic signs, et al.), which means possible missing of few factors affecting the latency. Thus, further population-based prospective studies are needed to fully clarify the clinical characteristics of PTE and factors affecting the latency of PTE.

## Conclusion

PTE is more common in males than in females, and peaks in patients aged 0 to 12 and 15 to 27 years old. Most patients with PTE suffered moderate-to-severe TBI, including single or multiple craniocerebral injuries. Generalized onset seizures were the most common seizure type. PTE latency ranged from 8 days to 20 years, and about half had a latency period shorter than 1 year, and more than 80% had a latency period shorter than 10 years. Patients who had TBI at the age ≥ 18 years old, suffered severe TBI followed by multiple surgical operations, experienced acute seizures, and had residual disability were more likely have shorter PTE latency.

## Data Availability

The datasets used and/or analyzed during the current study are available from the corresponding author on reasonable request.

## Abbreviations

PTE: Post-traumatic epilepsy
TBI: Traumatic brain injury
AEDs: Antiepileptic drugs
LPTS: Late post-traumatic seizure
ILAE: International League Against Epilepsy
EEG: Electroencephalogram
CT: Computed tomography
MRI: Magnetic resonance imaging
PD: Puncture drainage
DC: Decompressive craniectomy
CP: Cranioplasty
mRS: Modified Rankin Scale
PTS: Post-traumatic seizure
